# Detection and quantification of *Plasmodium falciparum* in human blood by matrix-assisted laser desorption/ionization time-of-flight mass spectrometry: a proof of concept study

**DOI:** 10.1186/s12936-023-04719-8

**Published:** 2023-09-26

**Authors:** Marius Ahm Stauning, Christian Salgård Jensen, Trine Staalsøe, Jørgen A. L. Kurtzhals

**Affiliations:** 1 Department of Clinical Microbiology, Copenhagen University Hospital - Rigshospitalet, Copenhagen, Denmark; 2 Centre for Medical Parasitology, Department of Immunology and Microbiology, University of Copenhagen, Copenhagen, Denmark

## Abstract

**Background:**

Matrix-assisted laser desorption/ionization time-of-flight mass spectrometry (MALDI-TOF) has revolutionized identification of bacteria and is becoming available in an increasing number of laboratories in malaria-endemic countries. The purpose of this proof-of-concept study was to explore the potential of MALDI-TOF as a diagnostic tool for direct detection and quantification of *Plasmodium falciparum* in human blood.

**Methods:**

Three different *P. falciparum* strains (3D7, HB3 and IT4) were cultured and synchronized following standard protocols. Ring-stages were diluted in fresh blood group 0 blood drawn in EDTA from healthy subjects to mimic clinical samples. Samples were treated with saponin and washed in PBS to concentrate protein material. Samples were analysed using a Microflex LT MALDI-TOF and resulting mass spectra were compared using FlexAnalysis software.

**Results:**

More than 10 peaks specific for *P. falciparum* were identified. The identified peaks were consistent among the three genetically unrelated strains. Identification was possible in clinically relevant concentrations of 0.1% infected red blood cells, and a close relationship between peak intensity and the percentage of infected red blood cells was seen.

**Conclusion:**

The findings indicate that the method has the potential to detect and quantify *P. falciparum* at clinically relevant infection intensities and provides proof-of-concept for MALDI-TOF-based diagnosis of human malaria. Further research is needed to include other *Plasmodium* spp., wildtype parasite isolates and to increase sensitivity. MALDI-TOF may be a useful tool for mass-screening purposes and for diagnosis of malaria in settings where it is readily available.

## Background

Malaria, caused by infections with *Plasmodium* parasites, are a leading course of sickness and death especially in endemic low- and middle-income countries [1]. In severe cases malaria can progress from mild symptoms to fatal outcome in a matter of hours and suspicion of malaria is thus an emergency where a proper diagnose is required without delay. To guide treatment, an ideal diagnostic test for malaria should both be able to discriminate between *Plasmodium* spp., and to quantify the level of infection, as indicated by the percentage of infected red blood cells (IRBC).

Over the years several diagnostic methods have been developed to increase diagnostic precision and shorten time-to-result. Especially the development of rapid antigen tests (RDTs), such as lateral flow immunoassays, have had great impact on anti-malaria programmes due to its simplicity in use and quick results. Unfortunately, all current diagnostic methods also have limitations. Widespread technologies, such as loop-mediated isothermal amplification (LAMP) and RDTs cannot quantify the level of IRBC and can only provide tentative species discrimination. Furthermore, both LAMP and RDTs are limited by false positive results after treatment due to circulating *Plasmodium* antigens and DNA fragments [2–5]. Microscopy of blood as described by Giemsa in 1904, therefore, still remains the Gold standard for detection of malaria [6, 7]. In trained hands, microscopy can provide excellent sensitivity and species discrimination. However, even in trained hands microscopy is time-consuming, and the diagnostic precision is highly operator dependent with risk of misidentification and wrong diagnosis with less experienced operators.

The purpose of this study was to explore if matrix-assisted laser desorption/ionization time-of-flight mass spectrometry (MALDI-TOF) may be a candidate for a novel diagnostic approach. MALDI-TOF generates a unique mass spectrum reflecting the size and charge of proteins in an organism. This spectrum is then compared to known reference spectra in large databases. MALDI-TOF technology has revolutionized routine identification of bacteria and is broadly recognized as an inexpensive, fast and robust analysis with minimal risk of operator bias [8]. Apart from the MALDI-TOF platform, only limited laboratory equipment is needed to run MALDI-TOF. Pure material can be analysed directly after application of a thin layer of matrix, while blood-borne organisms require a few purification steps typically involving lysis of erythrocytes, centrifugation, and pipetting. Several commercial companies offer MALDI-TOF platforms, matrix components and purification kits. Despite this, MALDI-TOF analysis has not yet been used as a diagnostic tool for *Plasmodium* infections in humans. The aim of this study was to provide proof-of-concept for both detection and quantification of *P. falciparum* in human blood and thus create a framework for development of a novel diagnostic tool.

## Methods

### Overall study design

This proof-of-concept study was based on bio-bank strains of *P. falciparum* and negative controls of uninfected blood. Both sample types were prepared and analysed using the same MALDI-TOF protocols and resulting spectra were compared to identify differences.

#### Culture of *Plasmodium falciparum * and negative controls

Frozen samples of *P. falciparum* previously identified as HB3, IT-4 and 3D7 strains were thawed, cultured, and synchronized using magnet activated cell sorting (MACS, Miltenyi biotec, Bergish Gladbach, Germany) following standard protocols [9, 10]. Negative controls were prepared using the same protocol but leaving out *P. falciparum*. Lifecycle-stage and % IRBC were followed with daily microscopy of Giemsa-stained blood smears. When cultures reached > 2% IRBC with a predominance of ring stages, cultures were centrifuged at 779 g for 8 min (1800 RPM, CryoFuge 5500i, LH-4000 75,006,478, Thermo Scientific, Waltham, MA, USA). After centrifugation, cultures were adjusted to a final haematocrit of 50%. Final exact % IRBC was determined by microscopy of Giemsa-stained blood smears. A total of 2000 red blood cells (RBC) were reviewed pr. blood-smear. Final samples were stored at 5℃ for a maximum of 2 days before sample preparation. All cultures were controlled for *Mycoplasma* contamination with MycoAlert Mycoplasma Detection kit (Lonza, Basel, Switzerland). Strain identity was controlled with an in-house polymerase chain reaction (PCR) using MSP2-IC1-, MSP2-FC27- and GLURP-specific primer pairs. To control for cross-contamination, negative controls were controlled by microscopy of Giemsa-stained blood smears. A total of 100 microscopy fields containing RBC monolayers were reviewed for each negative control.

### Sample preparation

To extract parasites from RBC, 250 µl blood was vortex mixed for 15 s with 250 µl 0.7% saponin in 140 mM phosphate-buffered saline pH 7.4 (PBS) and incubated 5 min at room temperature. After incubation 1500 µl PBS was added and samples were vortex mixed for 15 s and centrifuged for 2 min at 2579 *g* (6200 RPM, Minispin F-45-12-11, Eppendorf AG, Hamborg, Germany). After centrifugation, the supernatant was removed, and the lid and edges of the sample tube were carefully cleaned with a sterile cotton swap without touching the pellet. The pellet was then resuspended in 2000 µl PBS and samples were centrifuged for 1 min at 12,045 *g* (13.400 RPM, Minispin F-45-12-11, Eppendorf AG). The resulting pellet was spotted in a thin layer on clean steel target plates using a wooden toothpick and 1 µl matrix (alpha-cyano-4-hydroxycinnamic acid, Bruker Daltonics, Billerica, MA, USA) was applied to each sample spot. Samples were dried at room temperature before further processing. The sample preparation protocol was based on a method described by Baumeister et al. to separate intact *Plasmodium* parasites from erythrocytes [11].

#### Identification of *P. falciparum*

Samples were analysed using a Microflex LT MALDI-TOF (Bruker Daltonics) and FlexContol v.3.4 (Bruker Daltonics). The following settings were applied: Sampling range 1000–20,000 Da, laser power 30–50% of maximum, accumulation of 800 shots pr. spectra, movement pattern random walk, maximum 20 shots allowed pr. raster position, automated peak quality evaluation from 4000 to 10,000 Da, standard exclusion of the one largest peak in the mass range, minimum peak resolution 200 a.u., fuzzy control peptide mode with high signal intensity, and automated termination if 100 consecutive shots failed. Resulting spectra were visually evaluated in FlexAnalysis v.3.4 (Bruker Daltonics). Baseline subtraction and smoothing was applied once before visual inspection. Peaks found in *Plasmodium* samples but not in the negative controls were noted. As a quality control, spectra were further compared to the existing bacterial database with the standard built-in automated comparison tool. MALDI-TOF calibration and detector check was preformed according to the manufacturer’s instructions before initiating the study.

#### Quantification of *P. falciparum*

A culture of *P. falciparum* 3D7 was diluted in fresh blood group 0 blood drawn in EDTA from healthy subjects. Dilution was done with 50%, 25%, 12.5%, 7.5% and 4% of the original sample. For each dilution step negative controls were prepared with the same concentrations of cultured negative samples and fresh EDTA blood. At each dilution step parasitaemia degree was determined by microscopy of Giemsa-stained blood smears, as previously described. Samples were hereafter processed using the same protocols for extractions and MALDI-TOF analysis as previously described. Further, peaks intensity was determined for six unique *P. falciparum* peaks for each dilution step.

## Results

### Identification of *P. falciparum*

**Fig. 1:**
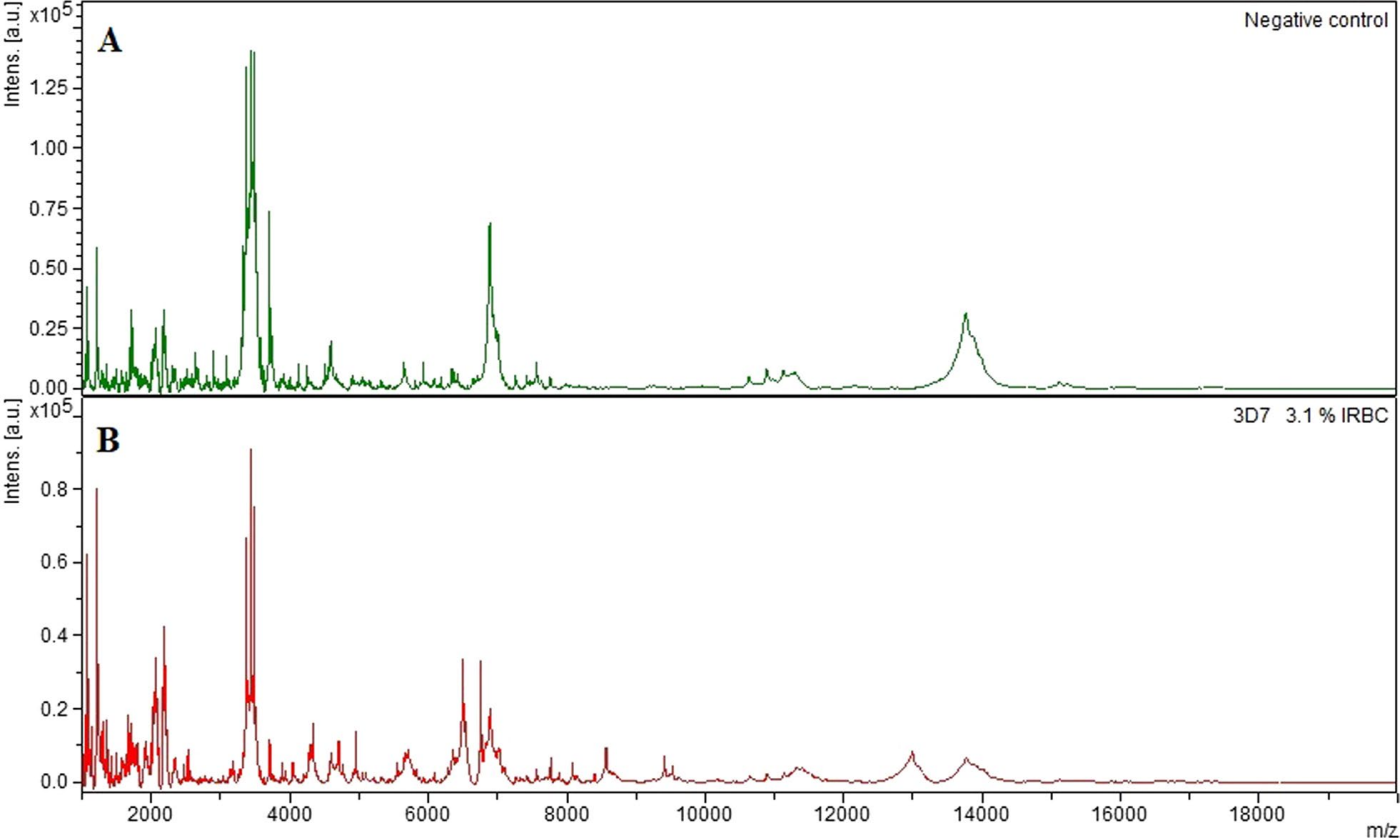
MALDI-TOF spectra of *Plasmodium falciparum *and negative controls. An example of mass spectra in the sample range 1000–20.000 m/z. A = Negative control of uninfected erythrocytes. B = Positive sample with 3,1% 3D7 *Plasmodium falciparum*-infected erythrocytes. Each peak in the spectra represents proteins of a specific charge and size. A full description of peak differences can be found in Table 1. Plasmodium cultures and negative controls were prepared using the same protocols. See Methods section for details

Visual inspection revealed 20 peaks that were specific for *P. falciparum.* The peaks were found in undiluted samples of all three strains. (Fig. 1; Table 1). Six additional *P.falciparum-*specific peaks were identified in the 3D7 strain, 1 peak in the HB3 strain, 1 peak in the IT4 strain, and 3 peaks in both the 3D7 and IT4 strain but not the HB3 strain (Table 1). After dilution of the 3D7 strain correct identification of *P. falciparum* samples was possible at concentrations down to 0.1% IRBC. In total All 18 extractions from uninfected RBC were correctly identified as negative. Of 23 *P*. *falciparum* extractions, 22 were correctly identified while 1 run was invalid (flatline - no peaks found). Subsequent re-run of this extraction was correctly identified.

**Table 1:** *Plasmodium falciparum*-specific peaks found by MALDI-TOF analysis

*Sample type*															
Culture type		3D7	HB3	IT4	NC	3D7	NC	3D7	NC	3D7	NC	3D7	NC	3D7	NC
% Culture		100	100	100	100	50	50	25	25	12,5	12,5	7,5	7,5	4	4
% EDTA dilution		0	0	0	0	50	50	75	75	87,5	87,5	92,5	92,5	96	96
*Microscopy results*															
Ring stages		54	49	30	0	32	0	13	0	7	0	4	0	2	0
Late stages		8	9	9	0	6	0	3	0	1	0	0	0	0	0
Total IRBC		3,1%	2.9%	2.5%	None	1,9%	None	0,80%	None	0,40%	None	0,20%	None	0,10%	None
*MALDI-TOF results*															
Successful runs		3 of 3	3 of 3	3 of 3	3 of 3	3 of 3	3 of 3	3 of 3	3 of 3	3 of 3	3 of 3	2 of 3	3 of 3	3 of 3	3 of 3
MALDI-TOF diagnose		Pos	Pos	Pos	Neg	Pos	Neg	Pos	Neg	Pos	Neg	Pos	Neg	Pos	Neg
*Peaks found (m/z)*															
1014		+	+	+	–	+	–	+	–	–	–	–	–	–	–
1130		+	+	+	–	+	–	+	–	+	–	+	–	+	–
1366		+	+	+	–	+	–	+	–	+	–	+	–	+	–
1470		+	+	+	–	+	–	–	–	–	–	–	–	–	–
1657		+	+	+	–	+	–	+	–	+	–	+	–	+	–
1984		+	+	+	–	+	–	+	–	–	–	–	–	–	–
2006		+	+	+	–	+	–	+	–	+	–	+	–	+	–
2473		+	+	+	–	+	–	+	–	+	–	+	–	+	–
2527		+	+	+	–	+	–	+	–	–	–	–	–	–	–
3176		+	–	–	–	+	–	+	–	–	–	–	–	–	–
4042		+	+	+	–	+	–	+	–	+	–	–	–	–	–
4300		+	–	–	–	+	–	–	–	–	–	–	–	–	–
4332		+	+	+	–	+	–	+	–	+	–	+	–	–	–
4421		–	+	–	–	–	–	–	–	–	–	–	–	–	–
4432		+	–	–	–	+	–	+	–	+	–	+	–	+	–
4704		+	–	–	–	+	–	+	–	+	–	+	–	–	–
4765		+	–	+	–	–	–	–	–	–	–	–	–	–	–
4773		–	+	–	–	–	–	–	–	–	–	–	–	–	–
4957		+	+	+	–	+	–	+	–	–	–	–	–	–	–
5547		+	+	+	–	+	–	–	–	–	–	–	–	–	–
5567		+	+	+	–	+	–	+	–	+	–	+	–	+	–
5705		+	–	–	–	+	–	–	–	–	–	–	–	–	–
6086		+	–	–	–	+	–	+	–	+	–	+	–	+	–
6286		+	–	–	–	+	–	+	–	–	–	–	–	–	–
6500		+	+	+	–	+	–	+	–	+	–	+	–	+	–
6533		+	–	+	–	+	–	+	–	+	–	+	–	–	–
6760		+	+	+	–	+	–	+	–	+	–	+	–	+	–
7886		+	+	+	–	+	–	–	–	–	–	–	–	–	–
8086		+	+	+	–	+	–	+	–	–	–	–	–	–	–
8568		+	+	+	–	+	–	+	–	+	–	+	–	+	–
8843		–	+	–	–	–	–	–	–	–	–	–	–	–	–
9340		–	–	+	–	–	–	–	–	–	–	–	–	–	–
9409		+	–	–	–	+	–	+	–	–	–	–	–	–	–
9530		+	–	+	–	+	–	–	–	–	–	–	–	–	–
9546		+	+	–	–	–	–	–	–	–	–	–	–	–	–
12,993		+	+	+	–	+	–	+	–	+	–	+	–	+	–

*Plasmodium falciparum*-specific peaks found by MALDI-TOF analysis using different parasite strains and dilutions. + indicates the presence of a peak at the given m/z. – indicates the absence of a peak at the given m/z. 3D7, HB3 and IT4 indicate the strains of *P. falciparum* used in the experiment. NC = Negative control cultures consisting of uninfected erythrocytes. IRBC = Plasmodium-infected red blood cells. EDTA dilution = fresh type 0 blood in EDTA drawn from healthy subjects. Giemsa-Stained blood smears were used for microscopy. A total of 2000 red blood cells were reviewed for microscopy of each *P. falciparum* culture. A total of 100 microscopic fields were reviewed for negative control cultures. Mass spectra were compared by visual inspection using the FlexAnalysis software (Bruker Daltonics, Bremen, Germany) see method section for details. Plasmodium cultures and negative controls were prepared using identical steps. See method section for details

### Quantification of parasitaemia

**Fig. 2:**
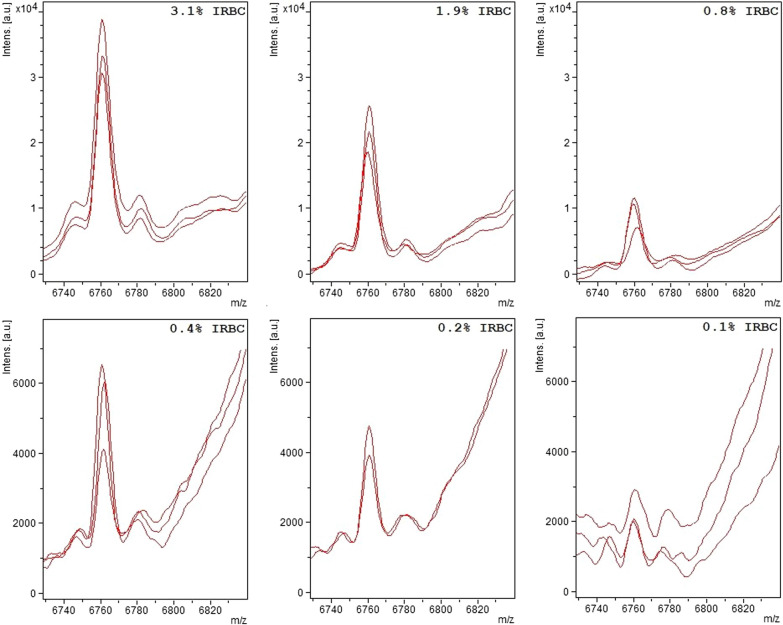
*Plasmodium falciparum *specific peak at 6760 m/z at different concentrations. The *Plasmodium falciparum*-specific peak at 6760 m/z in samples with different concentrations of infected red blood cells (IRBC). Each panel corresponds to a different concentration as indicated by the IRBC %. Traces from three independent sample preparations are shown for each concentration. Please note change in vertical scale between panels

**Fig. 3:**
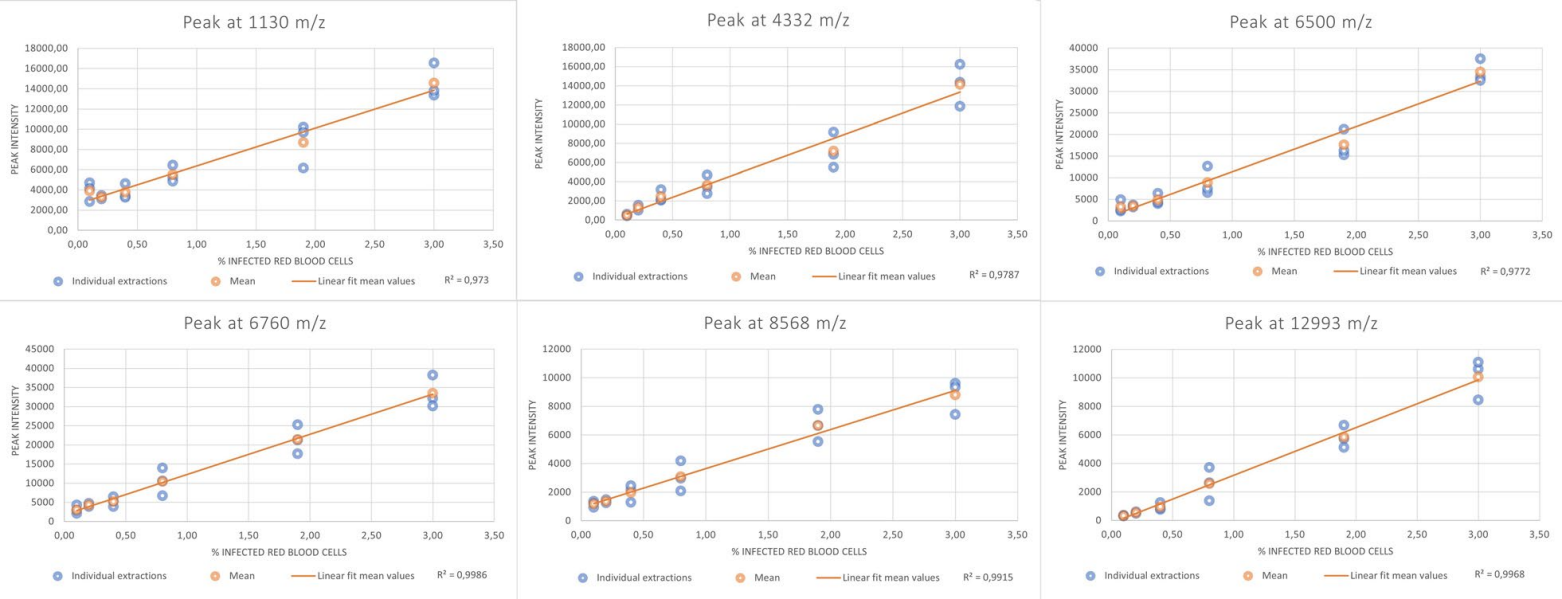
Correlation between peak intensity and % IRBC for different *Plasmodium falciparum *specific peaks. Correlation between signal intensity of the *Plasmodium falciparum* specific peaks at 1130, 4332, 6500, 6760, 8568 and 129,993 m/z and the concentration of *P. falciparu*m-infected red blood cells. Each panel represents a specific peak as indicated in the panel headline. Blue dots represent individual sample preparations at each concentration. Orange dots represent the mean peak intensity of the given concentration. The orange line represents a linear fit of mean peak intensity and concentration of *P. falciparum*-infected red blood cells

A linear relationship was seen between peak intensity and % IRBC with r^2^ ranging from 0.98 to 0.99 (Figs. 2 and 3). Furthermore, a higher number of *P. falciparum-*specific peaks were seen at higher concentrations of IRBC (Table 1).

### Quality controls

Genotyping confirmed strain identity as HB3, IT4 and 3D7. The control experiments did not suggest contamination of the samples. Thus, all cultures were *Mycoplasma* free, and automated comparison of spectra to the bacterial and fungal database showed a very low similarity with any microorganism in the database. Microscopy of negative controls did not identify any parasites.

## Discussion

### Findings in relations to existing literature

This study provide proof-of-concept for the use of MALDI-TOF for identification and quantification of *P. falciparum* in human blood. Despite the broad application in clinically microbiology, MALDI-TOF is not routinely used in parasitology [12]. Previous studies have provided early evidence of the capability of MALDI-TOF to identify different *Giardia, Cryptosporidium, Blastocystis, Entamoeba, Leishmania, Trichomonas, and Trichinella* species [12–14]. Like the current study, most previous studies have been done on cultured parasites and not with the use of clinical samples. For *Plasmodium* spp., previous attempts have been made to detect haemozoin using technologies similar to the MALDI-TOF used in the current study [15, 16]. These studies showed that haemozoin could be detected, but that the sensitivity was too low for clinical application [15–17]. Apart from the technical differences in these studies, a major difference is that the method used in the current study is based on a broad range of *P. falciparum* proteins instead of relying on a single residue product. Theoretically this approach can provide greater sensitivity and specificity than a hemozoin-based method. The haemozoin peaks have been reported around 500 m/z which is outside the detection area used in the current study [17, 18]. An interesting perspective of the current study is the possibility to quantify *P. falciparum*. Quantification is essential to inform patient management but currently microscopy is the only clinically applied method. Microscopy is time-consuming and several studies have shown large interobserver variation [7, 19]. In the current study both peak intensity and number of *Plasmodium*-specific peaks were found to correlate well with the concentration of *P. falciparum.* Both correlations could form basis for MALDI-TOF-based quantification of *Plasmodium* infections. Even if it should turn out to be impossible to improve the sensitivity of MALDI-TOF to a level where it can replace molecular based methods, a combination of MALDI-TOF quantification and nucleic acid detection of human *Plasmodium* could represent a major improvement for malaria diagnostics with respect to accuracy and time-to-result. Furthermore, MALDI-TOF-based quantification could potentially be used for treatment control, which is presently not possible with molecular methods or rapid diagnostic tests due to persistence of antigens and DNA fragments after treatment [2–5].

### Limitations of the study

This study was solely based on cultured biobank samples and only included *P. falciparum*. Further studies using clinical samples are required to examine the clinical application of the described method. Due to the design, using cultured *P. falciparum*, the studied samples presumably had a higher fraction of late-stage parasites than expected in clinical samples. To account for this, cultures were synchronized but late-stage parasites were still seen in control microscopy. It is unknown how this will have affected the peaks found by MALDI-TOF, but a recent comprehensive proteomic analysis of *P. falciparum* has shown a substantial overlap between proteins expressed by ring-stage and late stage parasites [20]. On the other hand, the reduced number of peaks found at low parasite concentrations could be due to disappearance of peaks specific to late-stage parasites after dilution. In this study spectra analysis was based on visual inspection. While this method is very useful in early-stage research, it is a limitation for clinical application of the method. In future studies it will be key to establish a database containing spectra from all *Plasmodium* species, as well as other blood borne parasites for automated spectra analysis and on-the-go differential diagnostics. Lastly, the reference method has been microscopy and results are, therefore, affected by the limitation previously described associated with this method. The effect is particularly important with regards to quantification, and it could be interesting to supplement future studies with quantitative-PCR or quantitation of parasite antigens for a more precise reference method.

### Future perspectives

Concentration limits for MALDI-TOF identification of blood borne bacteria are reported to be 10–1000 bacteria/µl [8]. From whole genome sequencing it has been calculated that two-third of the proteins expressed in *P. falciparum* are unique for the organism, a proportion that is higher than what is seen in other eukaryotes [21]. With that in mind, as well as the fact that *Plasmodium* parasites are considerably larger organisms than bacteria and, therefore, presumable have higher concentrations of proteins pr. organism, MALDI-TOF identification of *P. falciparum* should be possible at lower concentrations than the 0.1% IRBC used in this study. The direct cost of bacterial identification using MALDI-TOF is estimated to be less than 0.5 $ [22, 23]. When including depreciation of the machine, maintenance, and training, costs will vary depending on local factors such as salary levels, maintenance agreements, sample volume and estimated life-span of the MALDI-TOF platform, but overall costs have been estimated between 0.7 $ and 3.14 $ [23–25]. If a lower detection level could be achieved, it is possible that MALDI-TOF could allow cost effective malaria screening programmes. This is especially the case if MALDI-TOF proofs to be able to detect gametocytes, as this parasite stage is easily missed with microscopy and advanced PCR methods are needed for identification. Future studies focusing on enhanced extraction protocols, MALDI-TOF settings and matrix components are required to further explore these perspectives. If future research corroborates the applicability of MALDI-TOF in patient diagnosis and screening programmes, screening programmes could potentially be combined with vector control programmes. A growing number of studies have demonstrated that MALDI-TOF can distinguish between different species of *Anopheles* mosquitoes, and in 2017 Laroche et al. used MALDI-TOF to differentiate between *Anopheles stephensi* with and without *Plasmodium berghei* infection [13, 14, 26]. Another interesting perspective is the detection of resistance against anti-malarial drugs. Discrimination between bacterial strains with different resistance patterns based on characteristic peaks of the MALDI-TOF spectra is possible for a wide range of bacteria [27, 28]. Currently, no clinically relevant method for identification of anti-malarial drug resistance is established and anti-malarial drug resistance is, therefore, typically identified after treatment failure [29, 30]. The perspective of MALDI-TOF-based resistance testing would be targeted use of anti-malarial drugs and better surveillance of resistance development. Future studies are needed to explore this perspective. Finally, the peaks found in the MALDI-TOF spectrum represent proteins expressed by the *Plasmodium* parasite. Extended use of MALDI-TOF could identify proteins that are abundantly expressed and could guide studies using more advanced proteomics platforms to characterize these proteins further.

### Barriers for clinical use

This study should be seen as early-stage research and future research is needed to establish basic parameters such as sensitivity, specificity, and detection limits in a clinical population before clinical application. Secondly the detection limits will need improvement to compete with the sensitivity of the currently used methods, such as RDTs and microscopy. Detection limits for microscopy and RDTs vary depending on the manufacturer of the test and the skills of the microscopist [7, 31]. The World Health Organization estimates that the detection limits of microscopy under typical field conditions are 100 parasites per µL and recommends that RDTs are only used if they have > 75% sensitivity at 200 parasites per µL [7]. The 0,1% IRBC used in this study corresponds to 5000 parasites per µL and improved sensitivity is, therefore, needed. Lastly, the availability of MALDI-TOF platforms will be a barrier in many endemic countries. MALDI-TOF is considered a basic standard equipment for microbiology laboratories in high-income countries, and there is a growing availability of the equipment in low- and middle-income countries. Yet, MALDI-TOF is currently not available in many endemic settings. With the high establishing cost, cost-effective distribution of MALDI-TOF would require a general capacity building also involving its many applications in bacterial identification.

## Conclusion

Detection and quantification of *P. falciparum* in human blood with the use of MALDI-TOF is possible. Studies on clinical samples and novel sample processing protocols are however needed to further develop the method.

## Data Availability

The datasets used and/or analysed during the current study are available from the corresponding author on reasonable request.

## Abbreviations

EDTA: Ethylene Diamine Tetra Acetic acid
IRBC: Infected red blood cells
LAMP: Loop-mediated isothermal amplification
MACS: Magnet activated cell sorting
MALDI-TOF: Matrix-assisted laser desorption/ionization time-of-flight mass spectrometry
PBS: Phosphate-buffered saline
PCR: Polymerase chain reaction
RBC: Red blood cells
RDTs: Rapid diagnostic tests
RPM: Revolutions per minute
